# Workforce planning—going beyond the count

**DOI:** 10.1186/s13584-017-0179-7

**Published:** 2017-10-11

**Authors:** Lewis G. Sandy

**Affiliations:** Clinical Advancement, UnitedHealth Group, 9900 Bren Road E, Minnetonka, MN 55343 USA

**Keywords:** Workforce planning, International health comparisons, Physician shortage

## Abstract

Every country struggles with how best to meet the demand for health care services with the available resources. This commentary offers a perspective on the Israeli physician workforce and the analyses of Horowitz et al., which found age and gender differences in physician productivity and career longevity, differences across specialties, and a sizeable fraction of licensed Israeli physicians living abroad. Workforce planning can be subject to data collection and statistical uncertainties, but even more important are the assumptions and forecasts related to demand for services and organizational arrangements for care delivery. Readers should be cautious in analyzing productivity just by counting hours or years worked, and comparisons across countries may not account for differences in the nature of physician work. The question of whether Israel has enough physicians for the future has to go “beyond the count” to looking at the roles of other health professionals, the use of new technologies and new team configurations, and the overall efficiency and effectiveness of health care delivery systems such as hospitals, ambulatory care clinics, and community-based care.

Every country struggles with how best to meet the demand for health care services with the available resources. Given both the considerable cost and extended time needed to train physicians, assessment and tracking of physician workforce is a desirable element in any national health system. In a recent IJHPR article, Horowitz and colleagues report on analyses from 2008 and 2012 of the Israeli physician in workforce. [1] By linking national administrative files, the authors were able not only to assess the total “potential” physician workforce by tracking licensed physicians by specialty, they were also able to offer perspectives on the “effective” supply of physicians available, with a particular focus on physician productivity and physicians residing abroad.

The famous New York Yankee catcher and amateur philosopher Yogi Berra noted “It’s tough to make predictions, especially about the future”. Workforce planning has similar hazards. Not only are estimates of the supply and distribution subject to many data collection and statistical uncertainties, but the demand for services in the future may not resemble past or present circumstances. In the US, workforce planners in the 1990s forecast a surplus of physicians based on the productivity and organizational arrangements of group and staff model Health Maintenance Organizations (HMOs). [2] These forecasts significantly underestimated the growth of such organizational arrangements and the impact of changing demographics in the workforce, notably the changing gender composition and the generational preference for more employed practice settings. In a nutshell, they extrapolated from a generation of largely male physicians working 60+ hours per week vs. what actually developed, which was a workforce more 50:50 female/male and a generational preference for fewer work hours and better work/life balance. And the growth of group and staff model HMOs leveled off and care remained highly fragmented.

Given these caveats, how should Israeli health policymakers interpret the findings from Horowitz et als research? First, while this study is consistent with others noting gender differences in work hours and career longevity [3], research suggests that female physicians may have higher performance on quality of care metrics [4], have a more patient-centered communication style [5] and even positive effects on patient-level mortality risk [6], so one should be cautious in equating raw counts of work hours or years working with “productivity”.

Second, international comparisons of work hours should be interpreted with caution when the nature of the work across health systems may not be comparable. For example, the authors note that almost a quarter of US doctors work 61 h or more vs. 15% of Israeli doctors. Not accounted for in this comparison is the dramatically higher workload of US physicians devoted to administrative tasks related to billing and insurance and complex electronic health record systems [7].

Third, from a US perspective, it is notable that there are a fairly large proportion of licensed physicians practicing abroad. If this proportion remains stable and this cohort is seeking advanced training to bring back to Israel, then this (as the authors suggest) does not indicate a “brain drain”, but rather an investment in the future. However, if the proportion begins rising or evidence suggests a significant proportion of Israeli physicians are intending to emigrate after their training, those would be concerning trends. In either case, Israeli policymakers could benefit from better data on both supply of physicians and their competence in practice. In the US, periodic licensure requirements and associated surveys from some state licensing boards provide very granular data on physician supply, new regulations on health plans for provider directory data accuracy, and requirements by specialty Boards for ongoing Maintenance of Certification (MOC) provide a view of competence that is complementary to the use of quality metrics by hospitals and insurers. Serious consideration should be given to the development of similar databases in Israel.

The most important question of course in any workforce analysis is whether the health system had “enough” physicians. This study suggests that the Israeli physician workforce in aggregate is aging, and there are some specialties at greater risk for future shortages. The authors note that while these trends are concerning, there are strategies to address them, including expanding the use of multidisciplinary teams and expanding the pool of health professionals such as nurse practitioners (a relatively new field in Israel) [8, 9]. In both the US and Israel, innovations in health professions workforce and training hold great promise [10, 11]. Interprofessional Education and Practice (IPE/IPP) is a growing force in the US, and some states in the US are exploring an even wider range of health professional roles. Examples include programs to expand the role of pharmacists to help patients manage chronic diseases such as diabetes, leveraging the skills of Emergency Medical Technicians (EMTs) in community health assessment and management, and using lay Community Health Workers (CHWs) as active participants in health care teams.

Most health care delivery systems have far less “output” than what should be delivered given the resources available. Everything from poor scheduling systems to patient no-shows to bottlenecks in referrals—all conspire to degrade the overall system’s output, responsiveness and quality performance. In the US, operations research practices and management systems such as Lean (otherwise known as the Toyota Production System) [12] have been shown to dramatically improve performance, and one study in the US quantified how much primary care capacity could be improved through such techniques [13]. The Israeli health care system relies heavily on primary care, but is also wrestling with access to specialists and choice of physician. Addressing these issues will be important not only for the further evolution of the Israeli healthcare system, but also will have implications for the future workforce planning.

## Conclusion

This study adds new information for Israeli policymakers as they assess current and future medical education and workforce policy. Future studies should not only look at the numbers of physicians, but also at how they are deployed, how efficient the care delivery systems they work in are, and whether both process improvement and new technologies such as telehealth can get more out of the available workforce. These innovations, along with high performing teams, can deliver more care to individuals and populations. Also, team-based care can lead to higher professional satisfaction and less burnout. If newly-trained physicians can learn their craft as part of a high-performing team in a well-organized care system, then they will become maximally productive earlier and for longer. And if experienced physicians can leverage their local team and the broader delivery system to work a few more years vs. retiring early, that too can often be a win for the physician, the patient and the system. So while counting is important, so is going beyond the count.

## Abbreviations

CHW: Community health worker
EMT: Emergency Medical Technician
HMO: Health Maintenance Organization
IPE: Interprofessional education
IPP: Interprofessional practice
MOC: Maintenance of certification

## References

[1] Horowitz PK, Shemesh AA, Horev T (2017). Is there a doctor in the house? Availability of Israeli physicians to the workforce. Isr J Health Policy Res.

[2] Weiner JP (2002). A shortage of physicians or a surplus of assumptions?. Health Aff.

[3] Staiger DO, Auerbach DI, Buerhaus PI (2010). Trends in the work hours of physicians in the United States. JAMA.

[4] Reid RO, Friedberg MW, Adams JL, McGlynn EA, Mehrotra A (2010). Associations between physician characteristics and quality of care. Arch Intern Med.

[5] Roter DL, Hall JA, Aoki Y (2002). Physician gender effects in medical communication a meta-analytic review. JAMA.

[6] Tsugawa Y, Jena AB, Figueroa JF, Orav EJ, Blumenthal DM, Jha AK (2017). Comparison of hospital mortality and readmission rates for Medicare patients treated by male vs female physicians. JAMA Intern Med.

[7] Erickson SM, Rockwern B, Koltov M, RM ML, for the medical practice and quality Committee of the American College of physicians (2017). Putting Patients First by Reducing Administrative Tasks in Health Care: A Position Paper of the American College of Physicians. Ann Intern Med.

[8] Schoenbaum SC (2015). Issues relating to educating the future Israeli medical workforce: an international perspective. Israeli J Health Pol Res.

[9] Gamzu R (2016). Physician density planning in a public healthcare system: complexities, threats and opportunities—the case of the Israeli healthcare system. Health Policy.

[10] Aaron EM, Andrews CS (2016). Integration of advanced practice providers into the Israeli healthcare system. Isr J Health Policy Res.

[11] Schoenbaum SC, Okun S (2015). High performance team-based care for persons with chronic conditions. Isr J Health Policy Res.

[12] Toussaint J, Conway PH, Shortell S (2016). The Toyota production system: what does it mean, and what does it mean for health care? Health affairs blog.

[13] Green LV, Savin S, Lu Y (2013). Primary care physician shortages could be eliminated through use of teams, Nonphysicians, And Electronic Communication. Health Aff.

